# Unlearning implicit social biases during sleep: A failure to replicate

**DOI:** 10.1371/journal.pone.0211416

**Published:** 2019-01-25

**Authors:** Graelyn B. Humiston, Erin J. Wamsley

**Affiliations:** Department of Psychology and Program in Neuroscience, Furman University, Greenville, South Carolina, United States of America; University of Wuerzburg, GERMANY

## Abstract

A 2015 article in *Science* (Hu et al.) proposed a new way to reduce implicit racial and gender biases during sleep. The method built on an existing counter-stereotype training procedure, using targeted memory reactivation to strengthen counter-stereotype memory by playing cues associated with the training during a 90min nap. If effective, this procedure would have potential real-world usefulness in reducing implicit biases and their myriad effects. We replicated this procedure on a sample of n = 31 college students. Contrary to the results reported by Hu et al., we found no effect of cueing on implicit bias, either immediately following the nap or one week later. In fact, bias was non-significantly *greater* for cued than for uncued stimuli. Our failure to detect an effect of cueing on implicit bias could indicate either that the original report was a false positive, or that the current study is a false negative. However, several factors argue against Type II error in the current study. Critically, this replication was powered at 0.9 for detecting the originally reported cueing effect. Additionally, the 95% confidence interval for the cueing effect in the present study did not overlap with that of the originally reported effect; therefore, our observations are not easily explained as a noisy estimate of the same underlying effect. Ultimately, the outcome of this replication study reduces our confidence that cueing during sleep can reduce implicit bias.

## Introduction

Non-conscious biases are ubiquitous in social interactions, perpetuating discrimination even among people who do not explicitly endorse prejudiced beliefs [1–3]. For example, laboratory studies of hiring decisions demonstrate that participants who report no explicit racial bias nonetheless favour light-skinned candidates [4]. These implicit biases are insidious particularly because of their non-conscious, unintentional nature, as even persons with a strong implicit bias may not perceive any discrimination in their thoughts and actions, and may thus be unaware of their consequences [5]. Therefore, it is imperative to develop and disseminate procedures that effectively reduce these implicit biases and mitigate their impact on society.

A novel method of reducing implicit social biases was proposed in a 2015 *Science* paper [6], combining a computerized counter-bias training task [7] with “targeted memory reactivation” (TMR), a technique established to improve memory retention by boosting sleep-related consolidation. Sleep after learning is beneficial for memory [8–13], an effect which has been attributed to the iterative reactivation of recently formed memory traces in the sleeping brain [14–18]. An emerging literature has reported strong evidence that this reactivation of memory, which typically occurs spontaneously, can also be triggered externally by presenting sensory cues previously associated with the learning experience [18–21]. This TMR effect has been demonstrated using both olfactory [18] and auditory cues [19,20], and has been linked to the cellular-level replay of hippocampus-dependent memory in rodent models [21]. Thus, this technique shows promise as a practical method of enhancing memory and protecting it from subsequent forgetting [22].

Hu et al. [6] reported a novel attempt to use TMR to enhance memory for counter-bias training, with the prediction that TMR would strengthen the effect of the training and reduce implicit social biases. Participants were run in two groups, one of n = 21 and one of n = 19 several months later; their paper reported that results were similar across groups [6]. The training procedure involved first measuring implicit social biases toward Black people and women, using race and gender versions of the Implicit Association Test (IAT) [23]. Participants then completed a task that encouraged counter-bias thinking through responding to face-word pairings that contradict racial and gender stereotypes [7]. During this counter-bias training, two distinct sounds–one for the gender and one for the racial training–were played each time participants correctly affirmed a racial or gender counter-stereotype pairing. One of these two sounds (either the race-associated or gender-associated sound) was later played while participants were in slow-wave sleep (SWS) during a 90min nap, with the goal of reactivating their memory of the counter-bias training, and thus strengthening its effect [6].

Indeed, Hu et al. [6] reported that playing these sound cues during sleep strengthened the effects of counter-bias training. When implicit bias was tested again after the nap, the bias cued during the nap (race or gender) significantly decreased from prenap levels, while uncued bias remained unchanged. A long-lasting effect of this procedure would be particularly important evidence of the potential for practical application. Although the benefit of TMR was less apparent after a one-week delay, bias cued during the nap was still reduced in comparison to prenap levels. However, this bias reduction no longer differed significantly from that of the uncued bias type [6]. Still, overall, the study provided promising evidence for the efficacy of this novel method of reducing implicit social biases.

Yet rigorous science demands replication, especially for surprising findings that have potential real-world impact. In recent years, renewed attention has come to the value of replication in psychology, as large-scale efforts have demonstrated surprisingly low rates of reproducibility in the field [24,25]. For example, in a recent collaborative replication of 100 studies, of which 97 had statistically significant results (p < .05), only 36% of replications reached statistical significance, and 83% of replication effect sizes were weaker than in the original studies [24]. While a failed replication is never proof that an effect does not exist, as non-significant findings may be well within the range of possible outcomes when testing a real effect, the proportion of studies that failed to replicate in [24] is higher than what would be expected from sampling error alone. These and similar observations in recent years have highlighted the need to devote time and money to replication of important new findings before drawing strong conclusions [26,27].

Thus, because of our interest in the implications of effective implicit bias reduction during sleep, our lab conducted an exact replication of Hu et al. [6], repeating their procedure on a similar sample of college students, using materials provided by Hu et al. [6], and analyzing the data in the same manner. We expected to see a robust effect of cueing immediately after the nap, but were doubtful about our ability to detect an effect after a 1-week delay, given that the Cueing x Time interaction was non-significant at the 1-week delayed test in the original paper [6].

## Methods

### Participants and sample size

A target sample size of n = 30 useable participants was set by determining the number of observations needed to achieve power = 0.9 for detecting Hu et al.’s [6] originally reported effect of cueing (cued vs. uncued stimuli) on change in IAT score from before to after the nap (effect size *d*_*z*_ = 0.62, calculated from Hu et al.’s [6] original data). Enrollment continued until we reached the target number of qualifying participants.

Using the same criteria as Hu et al. [6], we excluded participants from analysis if they did not self-identify as white (n = 8 excluded) or male or female (n = 1 excluded). These non-qualifying participants were recruited early in the study with the goal of creating an exploratory comparison sample, but we later decided to stop enrolling non-white participants in order to complete data collection for the replication study in a timely manner. The sample of non-white participants was thus too small (n = 8) for meaningful statistical comparison. Descriptive statistics for this group are included in Table A in S1 Appendix. Participants were also excluded if they did not enter slow-wave sleep (SWS) during the nap (n = 6 excluded), or if they reported hearing the sound cue during the nap (n = 7 excluded). Following exclusions, there were n = 31 participants included in analysis (15 males, mean age of 19.55±1.23 SD, range 18–22; see Table 1). Participants signed written informed consent, and were compensated by receiving either $10/hr or credit for an introductory psychology course. The study was approved by the Furman University Institutional Review Board.

**Table 1:** Participant characteristics.

	*mean*	*±SD*
**Age (yrs)**	19.55	1.23
**ESS**	15.29	2.83
**SSS**	2.81	.75
**Baseline implicit bias**	.56	.41
**Prenap implicit bias**	.26	.48
**Postnap implicit bias**	.28	.46
**One-week delay implicit bias**	.40	.43
**Sex (% male)**	48%	
**Cue played during nap (% racial cue)**	54.8%	

Implicit bias values are the average D600 score for each timepoint.

### Procedure

**Fig 1:**
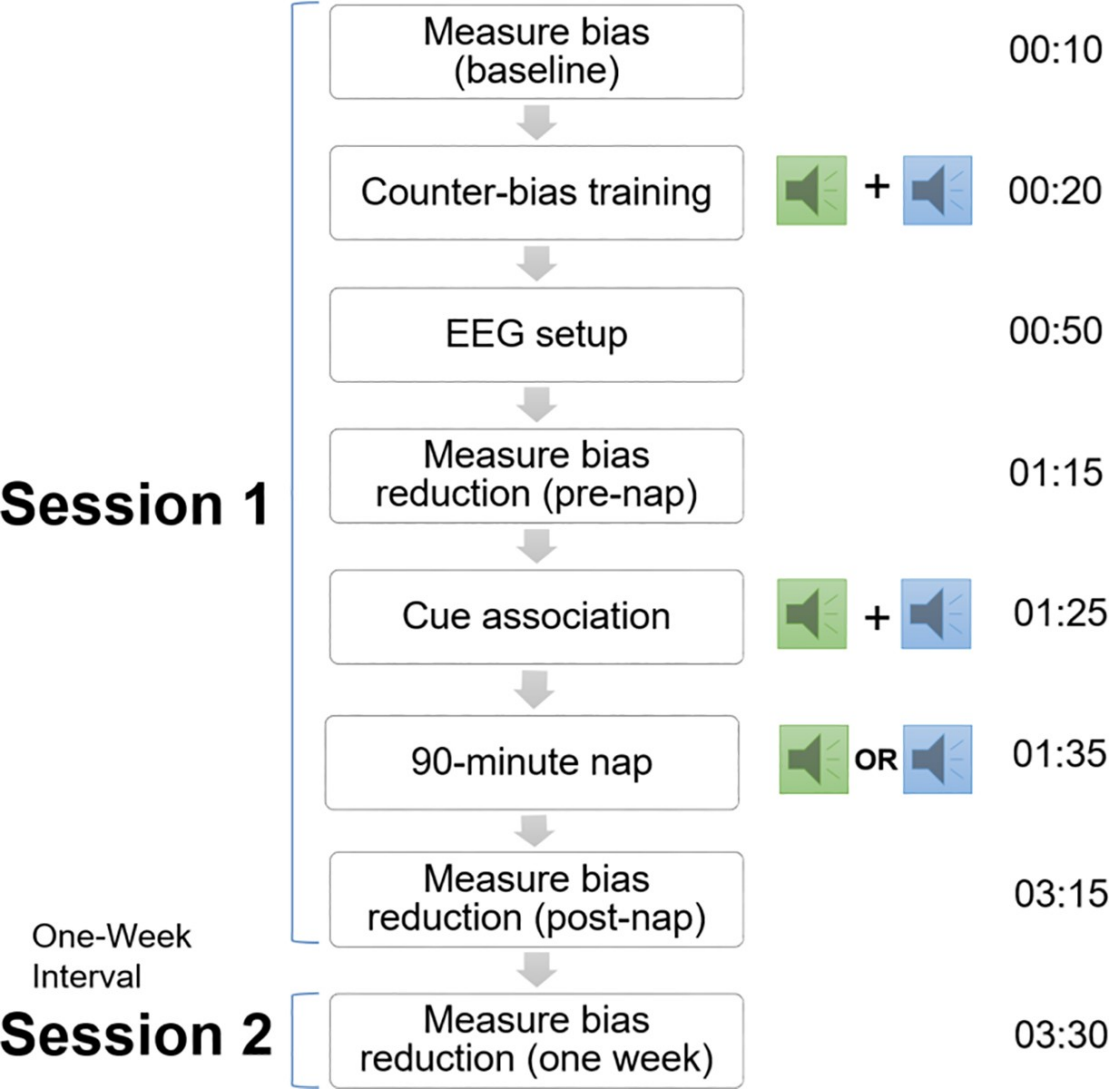
Experimental timeline. Participants completed implicit bias assessments both before and after a computerized counter-bias training procedure. TMR was then conducted during a 90min nap, and implicit bias was assessed again immediately following the nap. A final IAT and exit questionnaire were administered one week later. Times on right represent minutes elapsed. Green and blue sound icons represent the two distinct auditory cues associated with racial and gender counter-bias training, only one of which was presented during the nap TMR procedure.

The procedure followed that of Hu et al. [6] exactly (see Fig 1), with the exception of minor differences discussed below, including in the paperwork completed upon arrival at the laboratory, the timing of the post-nap inquiry about hearing the cue, an additional exit questionnaire at the conclusion of the study, and IAT order randomization. The procedure began between 10:00am and 1:00pm, and lasted for 3.5hrs. To facilitate the nap, participants were instructed to wake up at least two hours in advance of the study time. Upon arriving at the laboratory at the start of the procedure, participants completed demographics questionnaires, which gathered information about sex, race, sleep habits, and medications; the Epworth Sleepiness Scale (ESS; a measure of trait sleepiness) [28]; the Stanford Sleepiness Scale (SSS; a measure of state sleepiness; n = 6 of the n = 31 included in analyses had incomplete SSS data; see Table 1) [29]; and two visual analogue scales rating alertness and concentration. Participants also completed an exit questionnaire at the conclusion of the study in which they described what they believed to be the purpose of the experiment, and were asked explicitly whether they had heard the sound cue during the nap (S1 File).

Following the initial forms, participants completed the baseline IATs, one testing implicit racial bias and the other implicit gender bias (see below). Participants then completed the counter-bias training (see below) [7], in which they pressed a button to affirm female faces paired with science-related words, and Black faces paired with positive words, and were instructed not to respond to other pairings. One sound cue was used as positive feedback for correct responses during the gender counter-stereotype training, and another distinct sound cue during the racial counter-stereotype training, with the assignment of the cues to bias type counterbalanced. The IAT tasks, the counter-bias training tasks, and the sound cues were provided to us by Hu et al. [6].

After the training was completed, six EEG electrodes were attached to the scalp (F3/4, C3/4, O1/2), referenced to the contralateral mastoid. Eye and chin electrodes were also applied, in order to facilitate sleep staging. Impedance was kept to <10kΩ and signals were digitally acquired at 400Hz.

Participants then completed the prenap IATs, followed by a sound-cue retrieval task (see below) in which they actively matched female faces to science-related words, and Black faces to positive words. Each trial displayed both a science-related word and a positive word, along with a picture of either a female face or a Black face, which was presented along with the corresponding sound cue. The participant then clicked and dragged the face over to the matching counter-stereotype word. The purpose of the sound-cue retrieval task was to cement the association between the counter-bias associations and the corresponding sound cues, thus facilitating the TMR procedure later [6]. This task was also provided to us by Hu et al. [6].

After this task, participants began the 90min nap, approximately 95min after the start of the procedure. They were directed to lie on the bed in the testing room, after which the experimenter turned the lights off and left the room. White noise was played from a speaker directly above the pillow at 38–40 dB SPL. At the onset of SWS, the experimenter began playing one of the two sound cues from the same speaker, also at 38–40 dB SPL. Each sound cue lasted 1sec and was played at 4sec intervals, and was discontinued if participants showed signs of awakening or entering another sleep stage. The cue played was randomly chosen to be either the one previously associated with race (n = 17) or gender (n = 14), and was counterbalanced across participants, so that one cue was assigned to gender and the other to race for half of the participants, and vice versa. An average of 323±29 SEM individual cues were presented to each participant.

Following the nap, participants were awakened and the electrodes removed. The experimenter asked in a casual manner whether participants had heard anything during the nap, in order to gauge whether participants heard the sound cue. Their response was recorded by the experimenter (S2 File). There was a 10min break before participants took the postnap IATs, after which they left the laboratory. This differed slightly from Hu et al. [6], in which the verbal inquiry about noise during the nap occurred after the postnap IATs.

Participants returned one week later for a second session in which they completed the IATs again, followed by the added exit questionnaire (S1 File). We included this questionnaire, which was not a part of Hu et al.’s [6] procedure, because we believed that some participants who heard the sound cue might not indicate so without being asked more explicitly. The questionnaire began with several open-ended questions about the purpose of the study, after which participants were asked if they heard the sound cue during the nap, and if they had predicted that the sound cue(s) would be played during the nap (S1 File). These responses were scored by two judges, blind to experimental condition and whether the sound cue had been played during the nap (n = 6 participants who did not enter SWS never had a sound cue played, and responses from these participants were also scored by the judges). Judges determined whether the response to each open-ended question referenced the sound cue. They also determined whether the response referenced the sound as something that could affect the participants’ thoughts, memory, performance, or biases. Interrater reliability was 100%.

#### Implicit Association Test (IAT)

The IAT [23] is designed to measure implicit bias by comparing the speed with which one responds to group-attribute pairings that align with vs. contradict a common stereotype. For example, implicit racial bias is demonstrated when participants are slower to respond to Black faces paired with positive words, relative to Black faces paired with negative words. The IAT version in this study [23] was comprised of seven blocks; the 4^th^ and 7^th^ were the critical test blocks, each comprised of 40 trials, while the other blocks trained and familiarized the participants with the stimuli and test layout, and contained 20 trials each.

**Fig 2:**
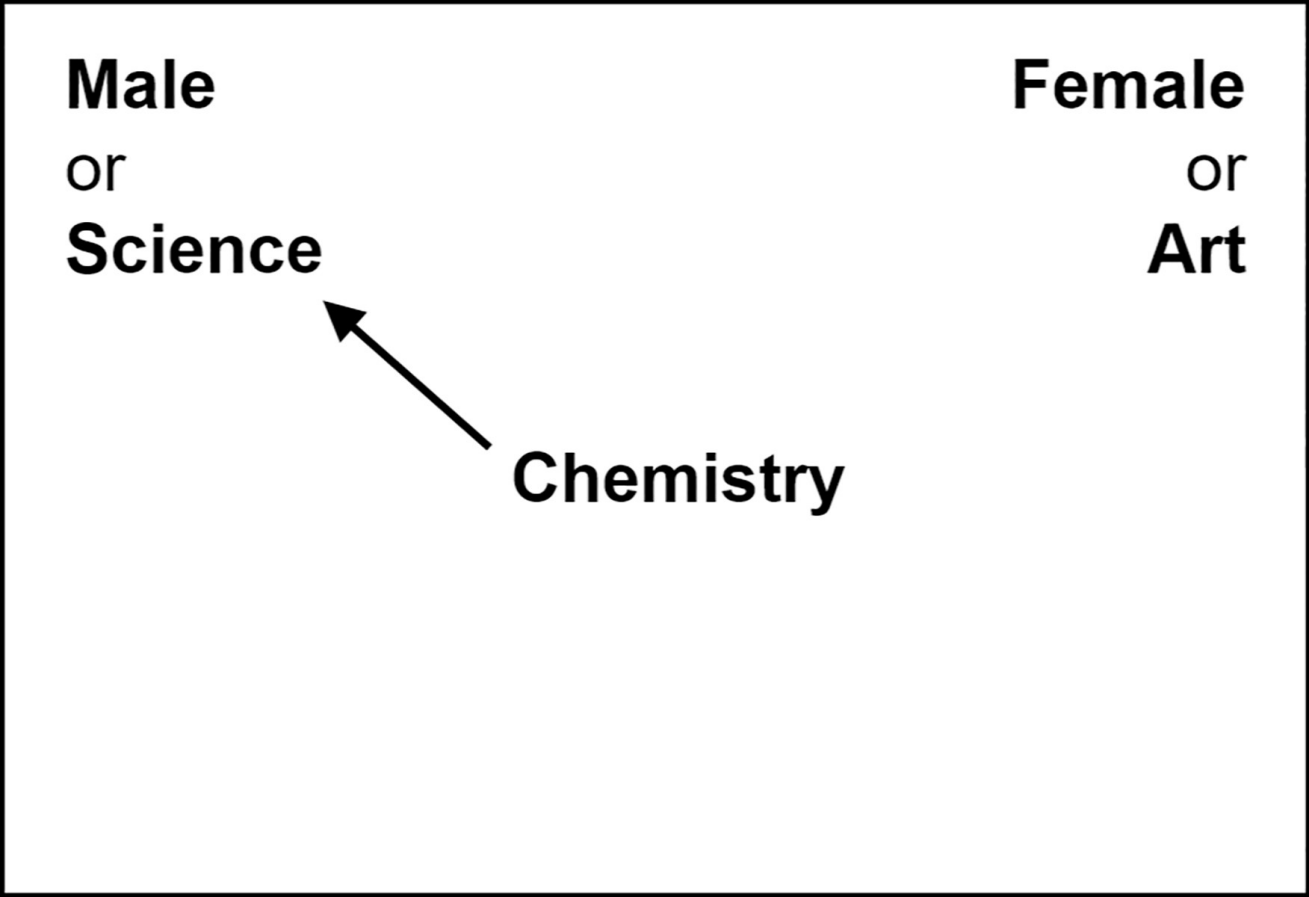
Implicit association test. Participants sorted a stimulus (word or picture) in the center of the screen into either the category in the top left or the category in the top right of the screen. For example, in this figure, the correct response would be to sort the word “Chemistry” to the left, as chemistry belongs in the “Science” category. Critically, categories at the top of the screen were comprised of one group (e.g. male or female) paired with one attribute (e.g. science or art). Implicit bias was measured by comparing the speed of sorting stimuli into *bias-congruent categories* (such as those pictured, where science is associated with male and art is associated with female) with the speed of sorting stimuli into *bias-incongruent categories* (such as male/arts and female/science).

Each test block trial required participants to sort a word or picture stimulus into one of two categories (see Fig 2). In the race IAT, the stimuli were positive or negative words (e.g. “sunshine” or “vomit”) and pictures of Black and White faces, with 10 of each type of stimulus. In the gender IAT, the stimuli were words related to arts or science (e.g. “Shakespeare” or “chemistry”) and pictures of male or female faces, also with 10 of each type of stimulus. The pictures used were chosen by Hu et al. [6] from the Eberhardt Lab Face Database, the NimStim Face Stimulus Set, and the Karolinska Directed Emotional Faces [30–33].

Participants sorted each stimulus into the appropriate category in the top left or right of the screen (Fig 2). Each category was comprised of a group and an attribute pairing: Black and White paired with “good” or “bad” in the race IAT, and male and female paired with “science” or “art” in the gender IAT. In the 4^th^ block, the group and attribute in each category were arranged to align with common biases (Black/bad and White/good, or male/science and female/art; Fig 2); in the 7^th^ block, category pairings contradicted common biases (Black/good and White/bad, or male/art and female/science).

Implicit bias was measured by comparing the speed of sorting stimuli into bias-incongruent categories (those in the 7^th^ block) with the speed of sorting into bias-congruent categories (those in the 4^th^ block). A participant who more quickly associates a female face with the word “art” than the word “science,” for example, demonstrates an implicit social bias. The mean response times from each IAT were converted into a *D*_600_ score, using the same algorithm reported by Hu et al. [6,34]. The order in which participants took the gender and race IATs at baseline was counterbalanced, and participants took the prenap, postnap, and delayed IATs in the opposite order of their baseline IATs. This differed slightly from the order randomization in Hu et al. [6], as reported in [6].

#### Counter-bias training

Participants completed one counter-bias training task for gender bias and one for racial bias, with order counterbalanced. Both counter-bias training tasks included 360 trials, with an intertrial interval of 1sec, completed in three blocks with breaks in between. In each trial, a picture of a face was presented with a word below it–a Black or White face paired with a positive or negative word in the race version, or a male or female face paired with a science- or art-related word in the gender version. In each version of the task, the target counter-bias trials (Black/positive and female/science) appeared 180 times; the remaining 180 trials were divided evenly between the other three possible pairings for that version. The words and faces used in the counter-bias training and the sound-cue retrieval task (see below) were chosen by Hu et al. [6] from the same sources [30–33] as were the words and faces used in the IATs, but were a different set that those used in the IATs.

For the gender counter-bias training, participants were instructed to press the spacebar if they saw a female face paired with a science-related word, and not to respond to any other pairing. Similarly, for the race counter-bias training, participants were instructed to press the spacebar only for the Black-positive pairing. Participants were instructed to respond as quickly and accurately as possible, in order to maximize the effect of the training [6]. A 1sec sound cue was played as positive feedback when participants correctly pressed the spacebar for female-science pairings, and another distinct 1sec sound cue was played as positive feedback for correct Black-positive responses; both at approximately 46 dB SPL. The assignment of each sound cue to gender and race counter-bias training was counterbalanced across participants. The sound cue files can be found in the Supplementary Materials for Hu et al. [6] at www.sciencemag.org/content/348/6238/1013/suppl/DC1.

#### Sound-cue retrieval task

Participants completed the sound-cue retrieval task after the second, prenap IAT. The purpose of the task was to strengthen the association between the sound cues and the counter-bias training, thus facilitating TMR during the nap [6]. There were six blocks of 20 trials each, with an interstimulus interval of 1sec. In each trial, a picture of a female or Black face was presented on the left side of the screen, the corresponding sound cue was played, and a randomly chosen and positioned (top or bottom) science-related word and positive word were presented on the right side of the screen. Participants were instructed to use the mouse to drag the picture of the face to the corresponding word (female-science or Black-positive), and to do so as quickly and accurately as possible.

## Results

### IAT comparisons

**Fig 3:**
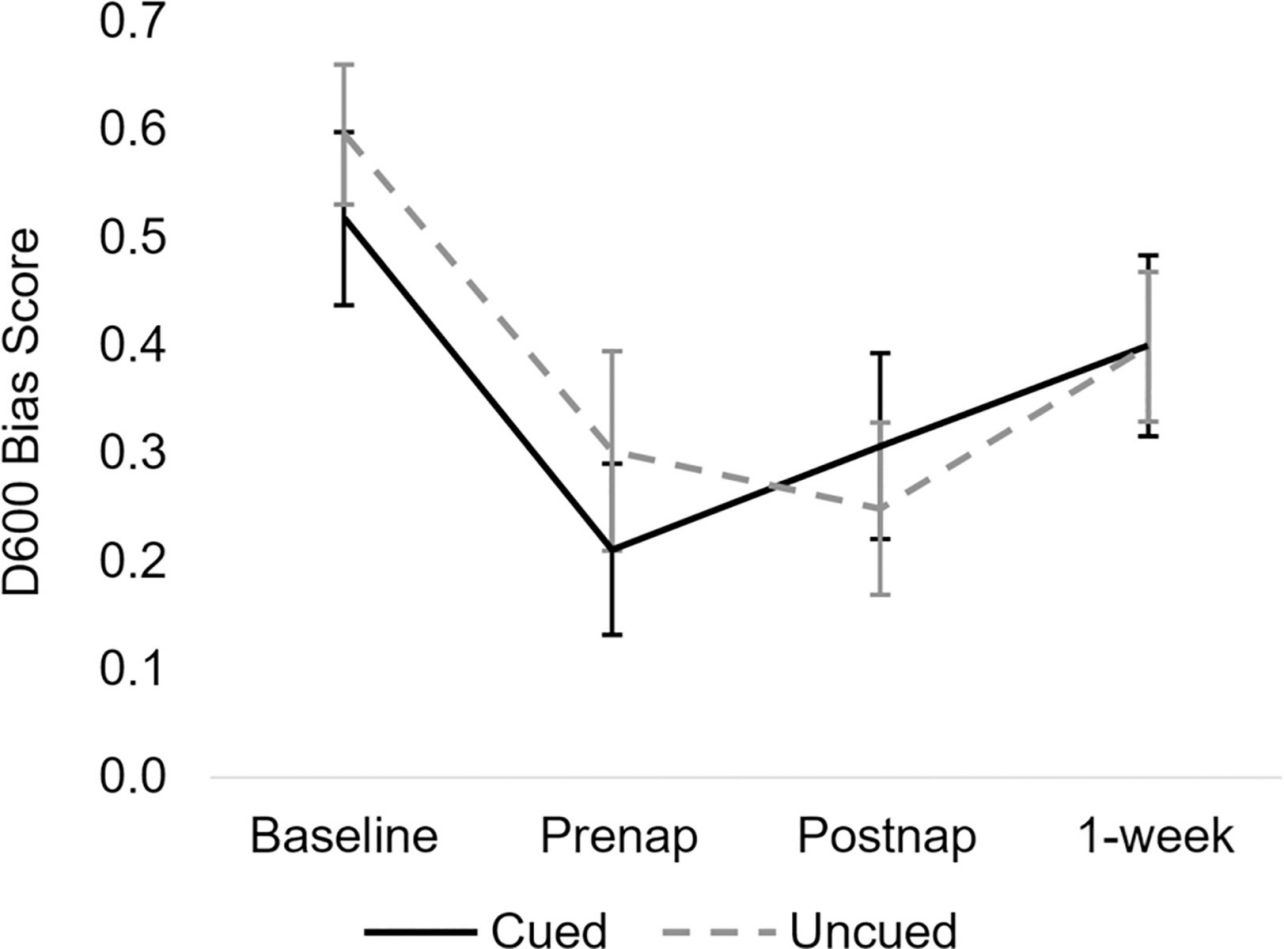
Average D600 scores at each IAT timepoint. Both cued and uncued bias significantly decreased from the baseline to the prenap IATs, with a non-significant increase in cued bias and decrease in uncued bias from the prenap to postnap IATs. Both cued and uncued bias non-significantly increased from the postnap to delayed IATs. The crucial Cueing (cued vs. uncued) x Time (prenap vs. postnap) interaction effect was not significant. Error bars ±SEM.

All data were analysed in the same manner as in Hu et al. [6]. Participants demonstrated implicit social biases on the baseline IATs, with scores for gender and racial bias significantly greater than zero (gender *t*(30) = 7.60, *p* < .001; race *t*(30) = 7.79, *p* < .001; see Table 2 and Fig 3). Bias levels were significantly reduced following counter-bias training (*F*(1,30) = 33.75, *p* < .001, *η*_*p*_^*2*^ = .53), from a mean IAT score of .56±.41 SD at baseline to .26±.48 SD at the prenap test (Table 1). There was an interaction between Bias Type (racial vs. gender) and Time (*F*(1,30) = 5.03, *p* = .03), such that although both racial and gender bias were reduced significantly from baseline to prenap, the reduction in racial bias (*t*(30) = 5.78, *p* < .001, *d* = .83) was larger in magnitude than the reduction in gender bias (*t*(30) = 2.47, *p* = .02, *d* = .49; Table 2)

**Table 2:** Race and gender implicit bias levels.

	Baseline		Prenap			
	*mean*	*±SD*	*mean*	*±SD*	*t*	*p*
**Race**	.62	.44	.20	.56	5.78	< .001
**Gender**	.49	.36	.31	.37	2.47	.02

Implicit bias values are the average D600 score for each timepoint.

**Fig 4:**
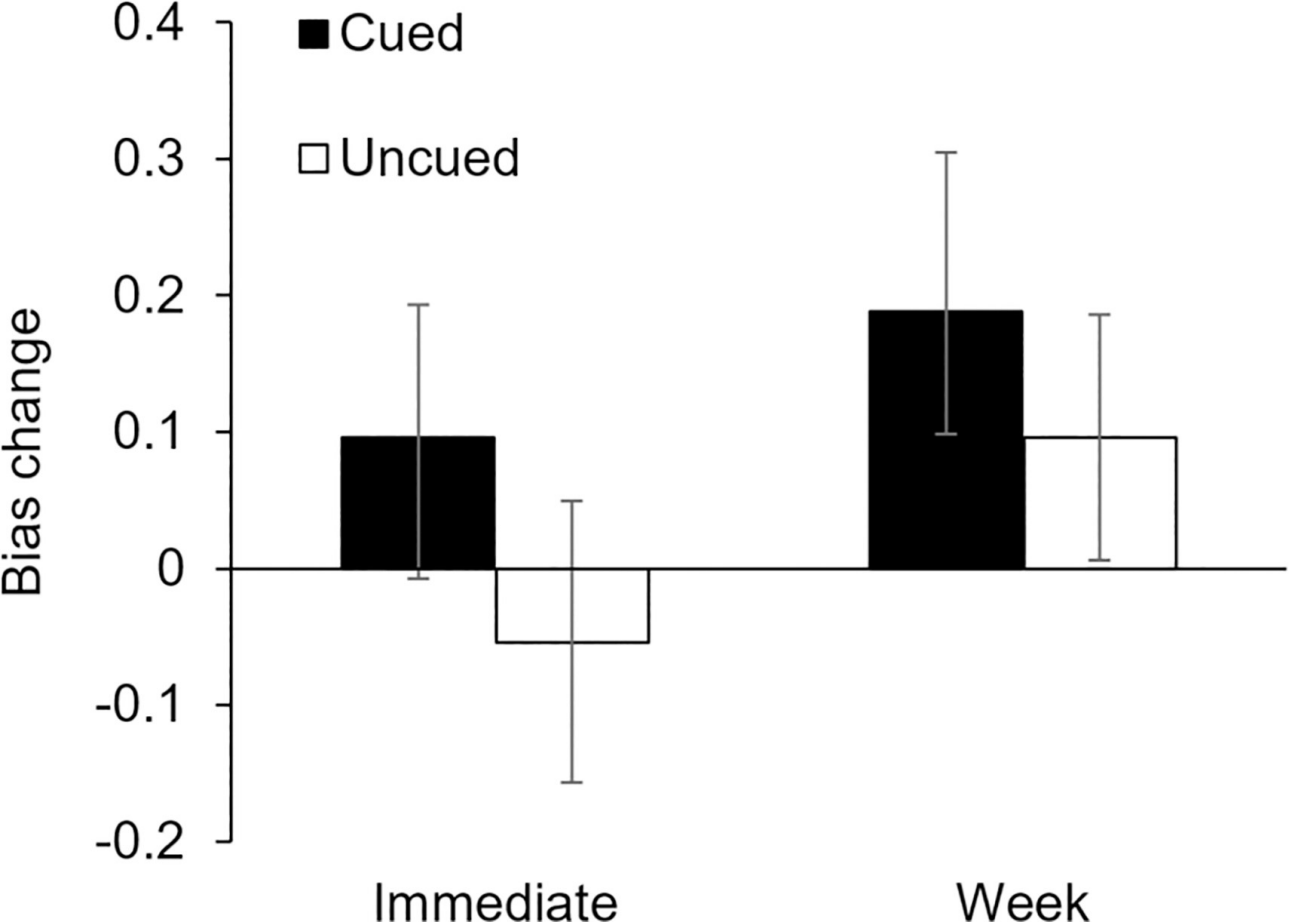
Change in implicit bias levels at the immediate and one-week delay tests. Cued and uncued bias did not change differentially from the prenap test to the postnap or 1-week delayed tests. Furthermore, cued bias *increased* numerically (though non-significantly) at both points and relative to uncued bias.

In contrast to Hu et al. [6], we found that cueing did not affect implicit bias change from the prenap to postnap test (Cueing x Time interaction: *F*(1,30) = 1.39, *p* = .25, *η*_*p*_^*2*^ = .044). This interaction remained non-significant when bias type was included as a factor in the model (Cueing x Time interaction: *F*(1,29) = 1.15, *p* = .29). While neither cued nor uncued bias changed significantly from prenap to postnap (cued: *t*(30) = .98, *p* = .33, *d* = .20; uncued: *t*(30) = .52, *p* = .60, *d* = .12), it should be noted that in contrast to the observations of Hu et al. [6], cued bias scores numerically (and non-significantly) *increased*, while uncued bias scores *decreased* (see Table 3, Fig 4).

**Table 3:** Implicit bias levels by condition.

	Cued		Uncued	
	*mean*	*±SD*	*mean*	*±SD*
**Baseline**	.52	.36	.60	.45
**Prenap**	.21	.51	.30	.44
**Postnap**	.31	.44	.25	.48
**1-week delay**	.40	.39	.40	.47

Implicit bias values are the average D600 score for each timepoint.

There was again no effect of cueing on the change in implicit bias from the prenap test to the 1-week delayed test (Cueing x Time interaction: *F*(1,30) = .61, *p* = .44, *η*_*p*_^*2*^ = .020), with both cued and uncued bias increasing non-significantly (cued: *t*(30) = 1.63, *p* = .11, *d* = .42; uncued: *t*(30) = 1.07, *p* = .29, *d* = .21; Table 3, Fig 4). Cueing also did not affect bias change from the baseline to delayed test (*F*(1,30) = .48, *p* = .49, *η*_*p*_^*2*^ = .016). Unlike the findings of Hu et al. [6], here uncued bias significantly decreased from the baseline to the delayed test (*t*(30) = 2.22, *p* = .034, *d* = .43), while cued bias did not significantly change (*t*(30) = 1.31, *p* = .20, *d* = .31; Table 3).

### Polysomnography

**Fig 5:**
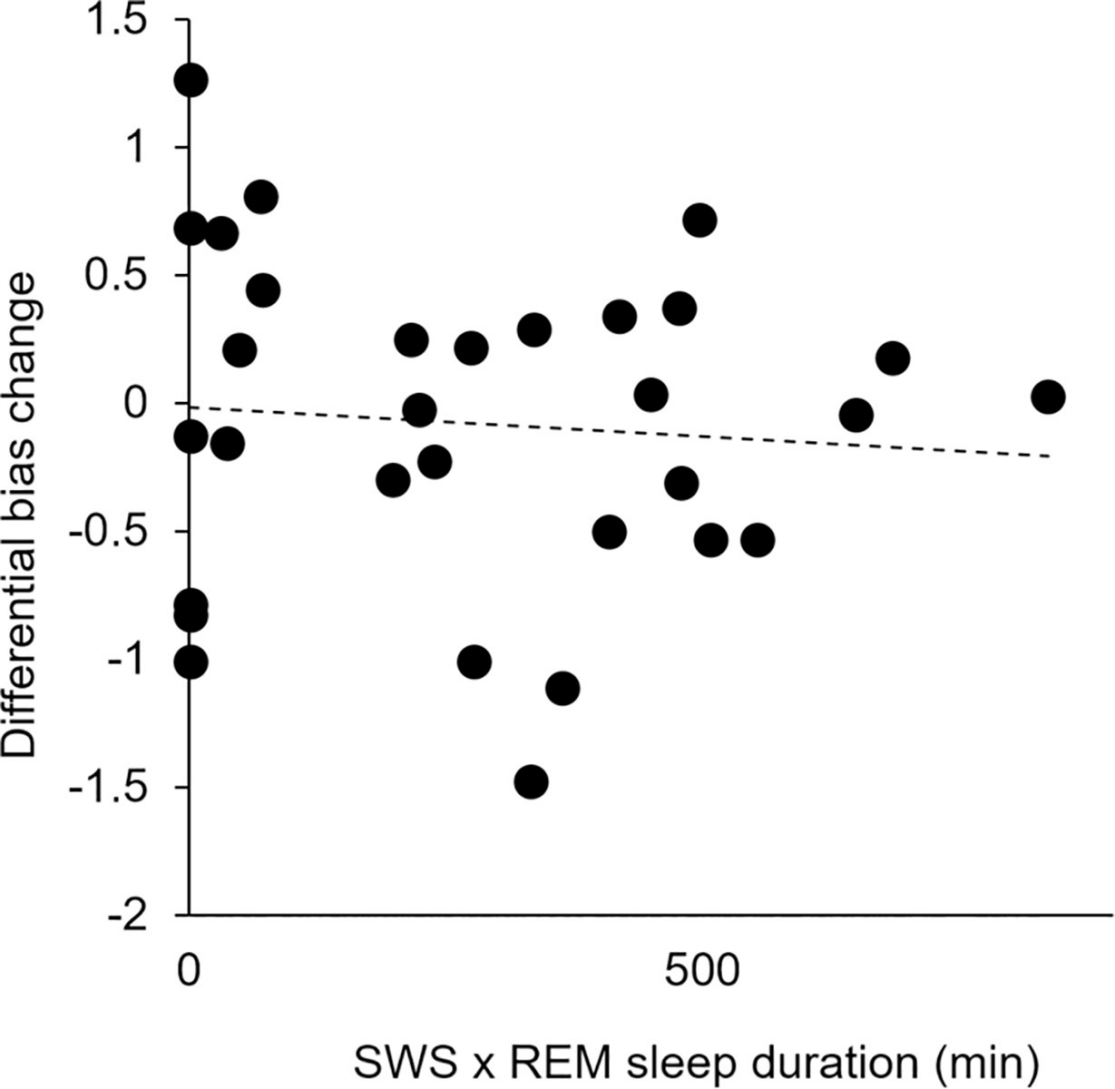
No association between minutes in SWS x minutes in REM and differential bias change.

In contrast to the observation of Hu et al. [6], we found that differential bias change (calculated as the baseline minus delayed score for uncued bias subtracted from the baseline minus delayed score for cued bias) did not correlate with the number of minutes spent in SWS multiplied by the number of minutes spent in REM (*r*(31) = -.09, *p* = .65; see Fig 5). The number of minutes spent in SWS and REM individually also did not correlate with differential bias change (SWS: *r*(31) = -.04, *p* = .85; REM: *r*(31) = -.13, *p* = .49). We performed additional exploratory correlations between differential bias change and the number of minutes the cue was played (*r*(31) = -.17, *p* = .37), the number of minutes spent in NREM1 (*r*(31) = .24 *p* = .20), the number of minutes spent in NREM2 (*r*(31) = .02, *p* = .94), and the total number of minutes spent asleep (*r*(31) = -.03, *p* = .87). None of these associations approached statistical significance. On average, participants spent 5.00±4.17 SD minutes in NREM1, 29.23±11.45 SD minutes in NREM2, 25.48±12.13 SD minutes in SWS (NREM3), and 11.52±7.95 SD minutes in REM (see Table B in S1 Appendix for comparison to sleep architecture reported by Hu et al. [6]).

### Verbal report and exit questionnaire

Immediately upon awakening, each participant was asked a verbal, non-leading question about whether they heard any noise during the nap; their response was recorded by the experimenter as either a “Yes,” a “No,” or “Maybe/unsure/unclear” (S2 File, Table 4). Participants were also explicitly asked in the final exit questionnaire (S1 File) whether they had heard the sound cue during the nap, with the option to choose one of three responses: “Yes,” “Not sure,” or “No” (Table 4). In the open-ended portion of the exit questionnaire, n = 2 participants mentioned a sound when asked if anything during the IAT or counterbias training indicated the purpose of the experiment to them, but no participants mentioned the sound cue in response to any other questions. None of the n = 31 participants in our analysis reported hearing the sound cue in either the verbal report or exit questionnaire. No verbal postnap response was recorded for n = 1 participant; we included this participant in our analyses because they chose “No” on the exit questionnaire. There were no participants who reported hearing the sound cue verbally, and then reported not hearing it on the exit questionnaire, and we are thus confident that the n = 1 participant whose verbal response was not recorded did not hear the sound cue during the nap.

**Table 4:** Sound cue reporting.

	Reported Hearing Cue on Verbal Report?		
Reported Hearing Cue on Exit Questionnaire?	No	Maybe	*Total*
No	26	2	*28*
Maybe	2	0	*2*
*Total*	*28*	*2*	*30*

Participants’ responses to the postnap verbal inquiry and to the exit questionnaire. A response was not recorded for n = 1 participant; this participant reported that they did not hear the sound cue on the final exit questionnaire.

## Discussion

In this replication study, we failed to find evidence that TMR strengthens the effects of counter-bias training, either immediately or after a 1-week delay. This decreases our confidence that TMR can be used to reduce implicit social biases.

**Fig 6:**
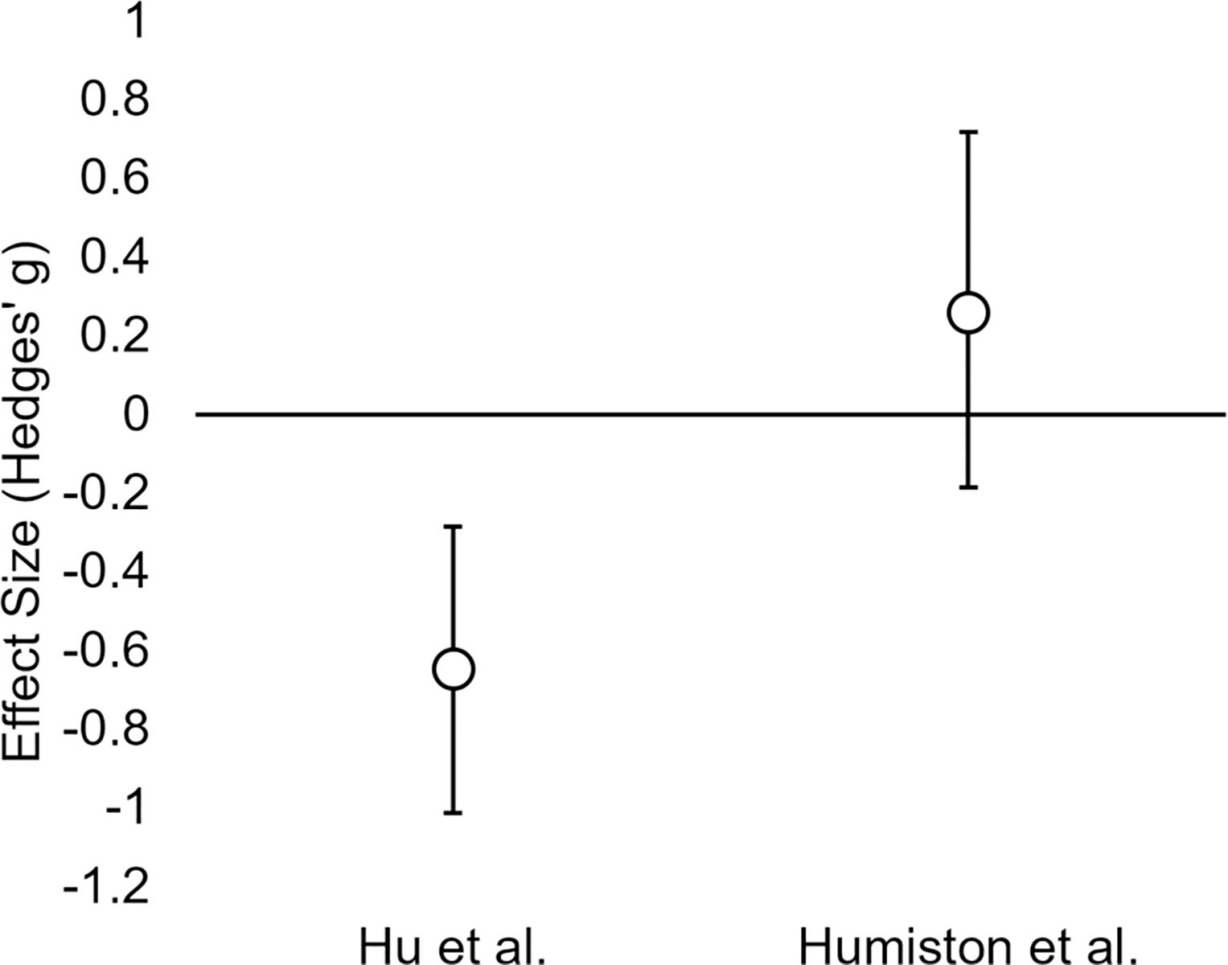
95% CIs for the immediate postnap effect of cueing in Hu et al. [6] and in our replication attempt. The confidence intervals do not overlap, suggesting that the two studies are not estimates of the same effect. However, our confidence interval overlaps with 0, allowing the possibility of a small effect (Hedges’ *g* = -0.18) in the direction observed by Hu et al. [6].

Importantly, this failure to replicate does not necessarily indicate that Hu et al.’s [6] report was a false positive–our failure to detect a cueing effect could be a case of Type II error. At the same time, there are several reasons why Type II error is not the most likely explanation for our results. First, the current replication was well-powered to detect an effect of the size reported by Hu et al. [6] (power of 0.9 to detect the originally reported effect of *d*_*z*_ = 0.62). While it is well known that published studies tend to overestimate the size of a true effect, in this case, if the true effect of cueing on bias reduction were even 25% smaller than that reported by Hu et al. [6] (*d*_*z*_ = 0.47), power of this replication study would still be 0.7. Second, the cueing effect we observed is in the opposite direction and is significantly different from the effect reported by Hu et al. [6] (Fig 6). Finally, although the 95% confidence interval of our non-significant effect is consistent with a very small decrease in cued bias (Hedges’ *g* = 0.19), the size of this effect is too small to have been reliably detected by either the original study or our replication. Taken together, these observations suggest that the current cueing effect is significantly different from that reported by Hu et al. [6], and unlikely to indicate simply a noisy estimate of the same underlying true effect.

There are some limitations of this study. First, at the outset of this study, a larger sample size would have been ideal. However, our 95% confidence interval is non-overlapping with that of Hu et al. [6] (Fig 6), encompassing only bias reductions too small to have been detected by the original study. This suggests that the precision of this replication is sufficient to draw meaningful conclusions [35]. Second, it is possible that there was a difference between our procedure or participant sample and that of Hu et al. [6] that influenced the results. There were a few minor procedural differences: The post-nap verbal inquiry about noise was asked immediately after the nap in our study, and after the post-nap IATs in Hu et al. [6]; and the participants in Hu et al. [6] were compensated through course credits, whereas in the current study n = 19 received course credits and n = 12 received a cash payment (Table A in S1 Appendix). Though it is possible that these differences could have influenced the results, there is no *a priori* reason to believe so; moreover, when taking compensation type into account, there was still no effect of cueing (prenap vs. postnap, Cueing x Time interaction: *F*(1,29) = .91, *p* = .35). Additionally, a larger portion of our participants entered REM sleep (81%, Table B in S1 Appendix) than did the participants in Hu et al. [6] (60%). Here, we note that an increased amount of REM in our sample would, if anything, have been expected to *increase* the effect of cueing, as REM is identified as a crucial mediator of the effect in Hu et al. [6].

Finally, we reiterate the possibility that our results could reflect an underestimation of a true effect that was overestimated in Hu et al. [6]. Although we do not consider this to be the most likely scenario, if this were the case, a meta-analytic summary effect derived from combining the two studies would be a useful guide as to the probable size of this effect. Using a random-effects model weighted by study precision, this summary effect is estimated to be quite small, at Hedges’ *g* = -.198. This effect would be difficult to detect in future studies, and arguably of little practical consequence in thinking about effective interventions to apply at the individual level.

Although we do not find evidence that TMR can make counter-bias training more effective, our data are somewhat consistent with the hypothesis that the computerized counter-bias training procedure employed in these studies is effective [7]. Implicit bias as measured by the IAT was reduced by 67% for race and 37% for gender after training, and was significantly reduced after one week (*η*_*p*_^*2*^ = 0.15). While this reduction in bias is consistent with an effect of the counter-bias training, it could also represent an unrelated effect of repeated testing on the IAT, as neither our current study nor Hu et al. [6] included a control group who was not provided with counter-bias training.

It may be possible to adapt or modify this counter-bias training procedure in other ways in order to make it more effective. However, recent research using counter-bias procedures to reduce implicit social biases has had mixed success, and has not demonstrated that such procedures can in turn reduce explicit biases or affect behavior [7,36–40]. In summary, this failure to replicate casts doubt on the claim that TMR could be used to strengthen counter-bias training and meaningfully reduce implicit social biases.

## Supporting information

S1 Appendix Additional descriptive statistics. Table A reports implicit bias levels based on demographics and compensation, and Table B compares sleep architecture for our study and Hu et al. [6].
(DOCX)

S1 File Exit questionnaire. The two-part final exit questionnaire given to participants at the conclusion of the second session.
(DOCX)

S2 File Verbal report. The form filled out by the experimenter to record each participant’s verbal responses to whether they had heard any noises during the nap.
(DOCX)
