# Supplementary material for: Unlearning implicit social biases during sleep: A failure to replicate
Source: PLoS One. 2019 Jan 25;14(1):e0211416. doi: 10.1371/journal.pone.0211416 (PMC6347202; doi:10.1371/journal.pone.0211416)
Supplement: S1 Appendix — Table A reports implicit bias levels based on demographics and compensation, and Table B compares sleep architecture for our study and Hu et al. [6]. (DOCX) [file pone.0211416.s001.docx]

**Table A. Implicit bias levels by race and compensation type.**

| **Implicit bias** | **Participant group** | | | | | | | |
| --- | --- | --- | --- | --- | --- | --- | --- | --- |
|  | Main sample^1^ (n=31) | | Non-White^2^ (n=6)^3^ | | Received course credits^1^ (n=19) | | Received cash^1^ (n=12) | |
|  | *mean* | *±SD* | *mean* | *±SD* | *mean* | *±SD* | *mean* | *±SD* |
| Baseline IAT cued | .52 | .36 | .36 | .63 | 0.59 | 0.39 | 0.40 | 0.30 |
| Baseline IAT uncued | .60 | .45 | -.22 | .58 | 0.61 | 0.45 | 0.58 | 0.46 |
| Prenap IAT cued | .21 | .51 | .04 | .32 | 0.31 | 0.53 | 0.06 | 0.47 |
| Prenap IAT uncued | .30 | .44 | .13 | .69 | 0.36 | 0.43 | 0.21 | 0.47 |
| Postnap IAT cued | .31 | .44 | -.46 | .41 | 0.45 | 0.48 | 0.08 | 0.26 |
| Postnap IAT uncued | .25 | .48 | .12 | .60 | 0.27 | 0.53 | 0.22 | 0.39 |
| 1 week IAT cued | .40 | .39 | .13 | .41 | 0.43 | 0.38 | 0.36 | 0.42 |
| 1 week IAT uncued | .40 | .47 | .17 | .23 | 0.44 | 0.49 | 0.33 | 0.45 |

Implicit bias values are the average D600 score for each timepoint. ^1^White participants only. ^2^Not included in main sample or replication analyses. ^3^Of the n=8 non-White participants who were recruited, n=2 were excluded because they failed to enter SWS or reported hearing the sound cue during the nap. Of the n=6 remaining, n=5 identified as “Black or African American”, and n=1 identified as “More than one race”.

**Table B. Comparison of sleep architecture across studies.**

| **Stage** | **Hu et al. (2015)** | | **Humiston & Wamsley** | |
| --- | --- | --- | --- | --- |
|  | *mean* | ±*SD* | *mean* | ±*SD* |
| Wake | 21.4 | 3.1 | 17.0 | 1.9 |
| NREM1 | 7.3 | 0.9 | 5.0 | 0.8 |
| NREM2 | 26.0 | 1.7 | 29.2 | 2.1 |
| NREM3/SWS | 27.2 | 2.4 | 25.5 | 2.2 |
| REM | 5.4 | 0.9 | 11.5 | 1.4 |

Numbers correspond to minutes in each sleep stage. Data for Hu et al. (2015) obtained from Supplementary Materials at [www.sciencemag.org/content/348/6238/1013/suppl/DC1](http://www.sciencemag.org/content/348/6238/1013/suppl/DC1).
