# Supplementary material for: Unlearning implicit social biases during sleep: A failure to replicate
Source: PLoS One. 2019 Jan 25;14(1):e0211416. doi: 10.1371/journal.pone.0211416 (PMC6347202; doi:10.1371/journal.pone.0211416)
Supplement: S1 File — The two-part final exit questionnaire given to participants at the conclusion of the second session. (DOCX) [file pone.0211416.s002.docx]

**Exit Questionnaire**

1. What do you believe may be the purpose of this experiment?

**________________________________________________________________________________________________________________________________________________________________________________________________________________________________________________________________________________________________________________________________________________**

1. What elements of the procedure, if any, stood out to you as indicative of the overall purpose of the experiment?

**________________________________________________________________________________________________________________________________________________________________________________________________________________________________________________________________________________________________________________________________________________**

**________________________________________________________________________________________________**

1. Was there anything during the Implicit Association Test or the Implicit Association Training that indicated the purpose of the experiment to you? If so, what?

**________________________________________________________________________________________________________________________________________________________________________________________________________________________________________________________________________________________________________________________________________________**

1. What role do you think the 90-minute nap played in the procedure?

**________________________________________________________________________________________________________________________________________________________________________________________________________________________________________________________________________________________________________________________________________________**

1. Was there anything during the nap that indicated the purpose of the experiment to you? If so, what?

**________________________________________________________________________________________________________________________________________________________________________________________________________________________________________________________________________________________________________________________________________________**

1. We played two sounds while you were doing the training to reduce social bias. We then played them when you were asleep. Did you hear these sounds **during the nap while you were sleeping?**
   1. Yes
   2. Not sure
   3. No
2. Part of the purpose of this study was to reactivate your memory of the bias-reduction training by playing these sounds during your nap, so that you would remember the training while you were sleeping. Did you guess or figure out that we were going to play these sounds while you slept?
   1. Yes, I knew
   2. I suspected it
   3. I’m not sure
   4. No, I didn’t know
