# Supplementary material for: Unlearning implicit social biases during sleep: A failure to replicate
Source: PLoS One. 2019 Jan 25;14(1):e0211416. doi: 10.1371/journal.pone.0211416 (PMC6347202; doi:10.1371/journal.pone.0211416)
Supplement: S2 File — The form filled out by the experimenter to record each participant’s verbal responses to whether they had heard any noises during the nap. (DOCX) [file pone.0211416.s003.docx]

1. Did the participant hear anything during the nap?
   1. Yes
   2. No
   3. Maybe
   4. They are unsure
   5. It is unclear from their response
2. Did they hear general lab noise?
   1. Yes
   2. No
   3. Maybe
   4. They are unsure
   5. It is unclear from their response
3. Did they hear the sound cue?
   1. Yes
   2. No
   3. Maybe
   4. They are unsure
   5. It is unclear from their response
4. Did they report anything else?
